# Successful Percutaneous Extraction of a WATCHMAN FLX Device From the Left Ventricular Outflow Tract

**DOI:** 10.1016/j.jaccas.2024.103186

**Published:** 2025-02-12

**Authors:** Evelyn Goodyear, Aaron Kunamalla, Enrico G. Ferro, Ronuk M. Modi, Andre D’Avila, Andrew H. Locke, David Liu, Roger J. Laham

**Affiliations:** a Department of Medicine, Beth Israel Deaconess Medical Center, Harvard Medical School, Boston, Massachusetts, USA; b Division of Cardiology, Beth Israel Deaconess Medical Center, Harvard Medical School, Boston, Massachusetts, USA; c Division of Cardiac Surgery, Beth Israel Deaconess Medical Center, Harvard Medical School, Boston, Massachusetts, USA

**Keywords:** aortic valve, anticoagulation, atrial fibrillation, occluder

## Abstract

**Background:**

Although rare, embolization of left atrial appendage occlusion (LAAO) devices carries a significant morbidity and mortality burden.

**Case Summary:**

An asymptomatic 77-year-old woman with inability to tolerate anticoagulation due to gastrointestinal bleeding presented for 45-day transesophageal echocardiography following LAAO with a Watchman device, which demonstrated incidental device migration to the left ventricular outflow tract (LVOT). Percutaneous extraction was performed using a novel technique with rat tooth/alligator forceps to successfully retrieve the Watchman from the LVOT using a transaortic approach.

**Discussion:**

LAAO device embolization is a rare, yet serious, complication. Retrieval from the LVOT is typically performed with cardiac surgery to avoid damage to the mitral valve. This case demonstrates that percutaneous Watchman device retrieval from the LVOT is technically challenging but can be safely performed in select cases to decrease the length of hospitalization and morbidity associated with surgery.

## Graphical Abstract

**Figure:**
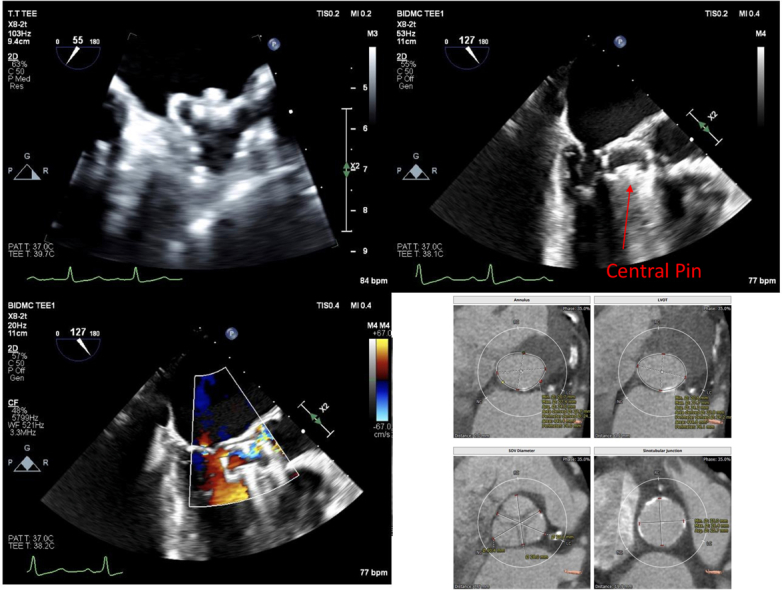

## History of Presentation

A 77-year-old female patient presented to the outpatient cardiology office for a routine 45-day post-implant transesophageal echocardiogram (TEE) following an uncomplicated 24-mm Watchman FLX (Boston Scientific) implantation at an outside hospital. The TEE showed that the Watchman device had migrated to the left ventricular outflow tract (LVOT). No effusion was present. The device appeared to be freely mobile but had not embolized through the aortic valve apparatus. She denied any symptoms. Physical exam was unremarkable with the exception of a III/VI crescendo–decrescendo systolic murmur auscultated over the left lower sternal border. There was no evidence of clinical heart failure or embolic phenomena.

**Take-Home Messages**

• We describe a novel percutaneous technique using rat tooth/alligator grasping forceps to retrieve an embolized Watchman device from the LVOT, which demonstrates a feasible alternative to surgical intervention for patients with LAAO device embolization.
• If this technique is selected, it is crucial to involve a multidisciplinary team for planning, intraprocedural expertise, and complication mitigation.

## Past Medical History

The patient had a cardiovascular history of hypertension, hyperlipidemia, coronary artery disease status post coronary artery bypass grafting (CABG), and atrial fibrillation. The rest of her medical history included remote non-Hodgkin lymphoma, esophageal achalasia, and lower gastrointestinal bleeding due to arteriovenous malformations requiring hospitalization and blood transfusions, for which the patient was recommended to undergo elective Watchman device implantation. She was on long-term apixaban (Bristol Myers Squibb) with her last dose on the day of her 45-day TEE.

## Differential Diagnosis

Though in this case the Watchman device was observed in the LVOT, embolized left atrial appendage occlusion (LAAO) devices have also been reported in the cavity of the left ventricle (LV), the left atrium (LA), and the aorta.^1^ The most common location of embolization is the aorta and LV.^1^

## Investigations

**Figure 1:**
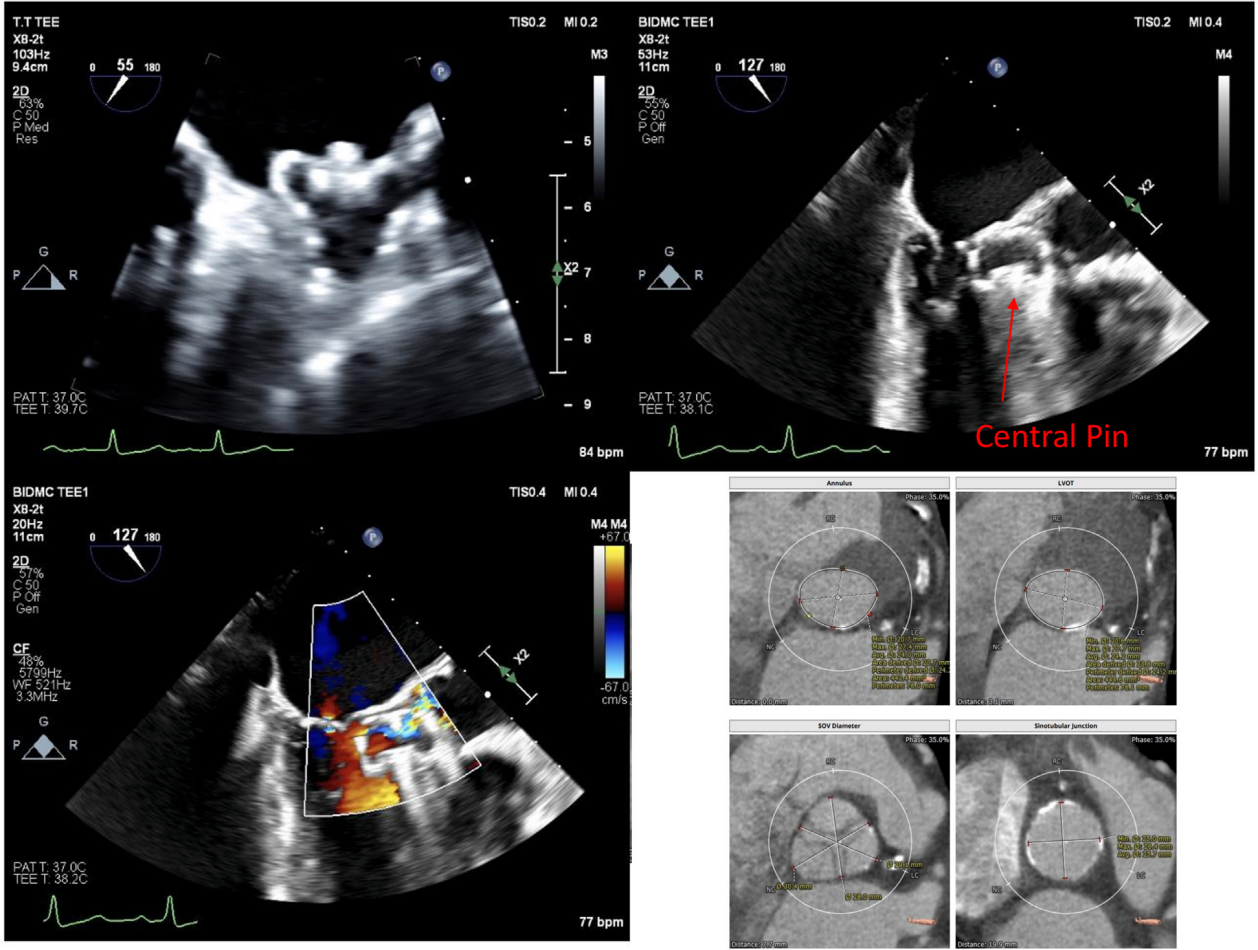
Pre-Intervention Imaging (Top left) Outside hospital implantation transesophageal echocardiogram (TEE) at 45° demonstrating a well-seated 24-mm Watchman FLX device within the left atrium in the left atrial appendage with postcompression measurements of ∼20 mm (17% compression). (Top right) Intraoperative extraction TEE at 130° demonstrating migrated Watchman device with central pin (arrow) toward the ventricular septum in the left ventricular outflow tract (LVOT) with (bottom left) color Doppler showing aortic regurgitation with flow through the Watchman and trivial mitral regurgitation. (Bottom right) 3Mensio (Pie Medical Imaging) report of the aortic valve measuring the annulus, LVOT, sinus, and sinotubular junction. LC = left coronary cusp; NC = noncoronary cusp; RC = left coronary cusp.

Outside hospital records and imaging from the index implantation were reviewed. Implantation TEE images revealed a left atrial appendage (LAA) with a chicken wing morphology. Based on TEE measurements, the ostium of the LAA measured 18 mm × 20 mm. A 24-mm Watchman FLX was selected for implantation. LAA ejection velocity was measured to be 20 cm/s. Following an uncomplicated transseptal puncture, the device was deployed in the LAA with 16% compression. An optimal tug test was demonstrated. All implantation criteria were met, and the device was deployed without issue (Video 1). It is important to note that the patient was in sinus rhythm during the time of the implantation. The 45-day TEE was reviewed, which demonstrated the Watchman FLX device was located in the LVOT, perpendicular to the aortic valve with the proximal end screw adjacent to the anterior mitral valve leaflet (Figure 1). The device had not embolized into the aorta, which we theorized was due to the presence of known moderate aortic stenosis. The Watchman device central pin was oriented towards the ventricular septum. On color Doppler, there did not appear to be aliasing through the LVOT, thus suggesting no significant LVOT obstruction. TEE was otherwise remarkable for small residual defect on the interatrial septum at the site of prior transeptal puncture with small left-to-right shunting as well as a patent foramen ovale.

Upon presentation to our institution, the patient’s vitals were closely monitored, and she remained hemodynamically stable while awaiting intervention. She was found to be in atrial fibrillation. Recent cardiac computed tomography (CT) was also reviewed, and aortic valve morphology was evaluated for possible transaortic valve replacement, if necessary, during Watchman retrieval (Figure 1).

## Management

**Figure 2:**
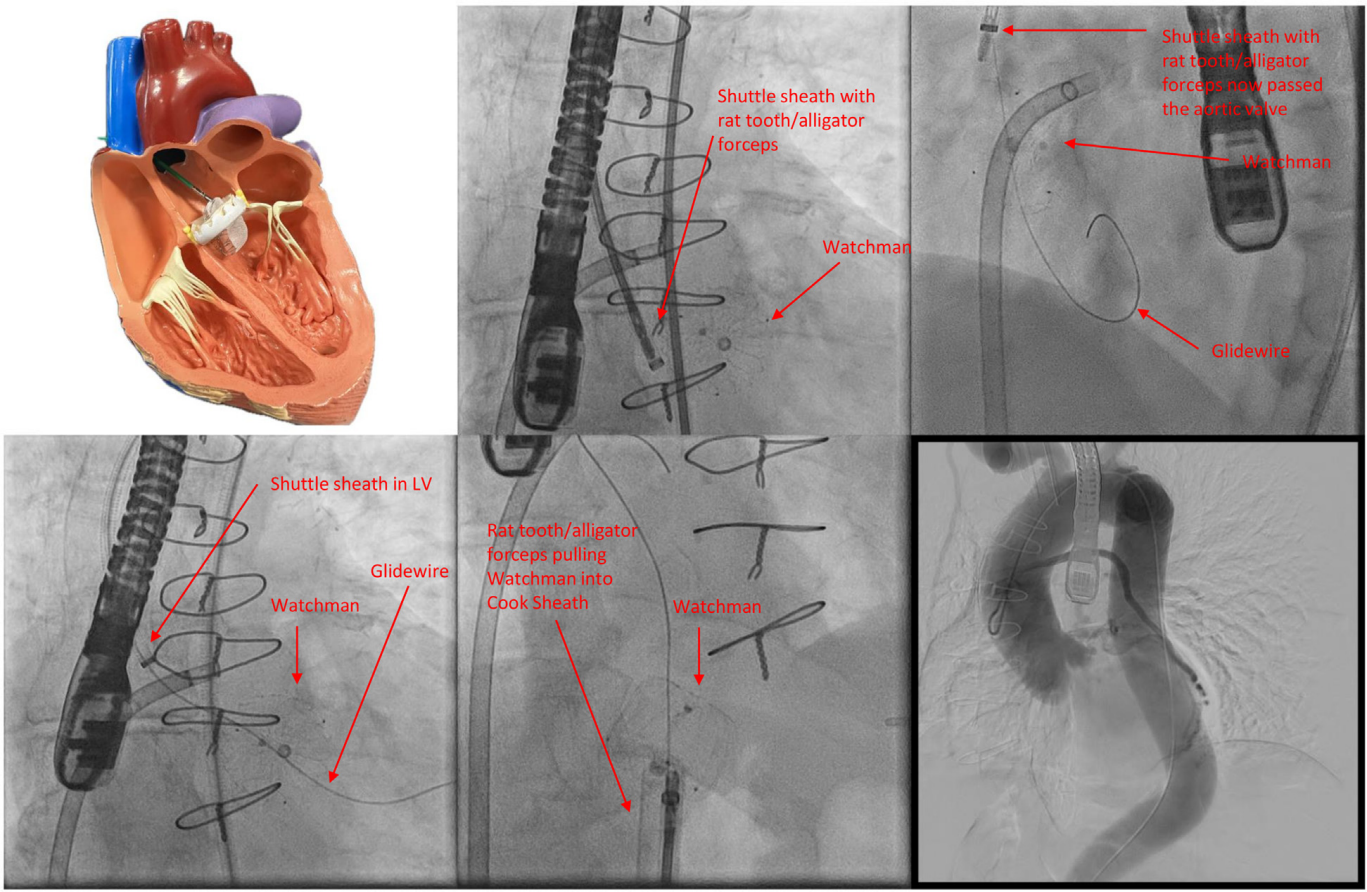
Sequence of Intervention (Top left) In vitro simulation of rat tooth/alligator forceps elongation of Watchman device. Top middle: Agilis NxT Steerable Introducer sheath advanced into the left atrium through the previous iatrogenic atrial septal defect. The shuttle sheath was positioned distal to the aortic valve, and rat tooth/alligator grasping forceps were advanced into the sheath (arrow). (Top right) The aortic valve was crossed using a straight glidewire (arrow). Bottom left: The shuttle sheath was carefully advanced into the left ventricle (LV) (arrow). The glidewire (arrow) was passed through the device mesh (arrow) in order to center the sheath over the device to facilitate grasping of the device without movement. (Bottom middle) Rat tooth/alligator grasping forceps (arrow) were used to grasp the Watchman (arrow) and pull the device through the aortic valve. The device was pulled into the descending aorta. An additional rat tooth/alligator grasping forceps was used to secure the Watchman. Both grasping forceps and Watchman were pulled into the Cook sheath. Bottom right: An aortogram was performed that did not demonstrate any complications.

Multidisciplinary preoperative planning was performed with combined efforts of structural cardiology, cardiac surgery, electrophysiology, interventional radiology, and vascular surgery. Given the patient’s age, history of CABG, and reluctance for surgery, the team recommended percutaneous intervention. Both retrograde and anterograde options were discussed. Given the proximity of the device to the aortic valve, a transaortic approach was ultimately selected as the primary extraction approach with simultaneous left atrial access via transseptal approach in the event of retrograde device migration back into the LV. Bench top testing with a Watchman device was performed to demonstrate that the device would be able to be pulled through a stenotic aortic valve using a rat tooth/alligator grasping forceps (Figure 2, Video 2).

The procedure was performed in a hybrid operating room under general anesthesia, guided by TEE. Specialists from structural cardiology, electrophysiology, cardiac surgery, and cardiac anesthesia were all present at the time of the procedure. A cardiac bypass pump team was placed on standby, and equipment was present to perform an urgent sternotomy or an urgent transcatheter aortic valve replacement procedure if needed. Before access, baseline TEE continued to show the Watchman device lodged in the LVOT, mobile during systole, with restricted opening of the aortic valve and Doppler showing flow through the aortic valve (Figure 1, Video 3). Under ultrasound guidance, an 8-F sheath was placed in the right femoral vein, while 6-F sheaths were placed in the right and left radial arteries and right femoral artery. Bilateral Sentinel Cerebral Protection Systems (Boston Scientific) were attempted to be placed but ultimately aborted due to severe tortuosity in the brachiocephalic artery. Additionally, the left subclavian artery was occluded.

An Agilis NxT Steerable Introducer sheath (Abbott) was advanced into the right atrium and advanced over a wire through the previous iatrogenic atrial septal defect into the LA (Figure 2). The Agilis sheath was left in the LA for the remainder of the case as a precaution in the event the Watchman device needed to be retrieved in an anterograde fashion through the mitral valve apparatus into the LV. Access was upsized to an 8-F sheath, which was then exchanged over an Amplatz-Extrastiff wire for a 20-F Cook 40 cm sheath (Cook Medical). An 8-F shuttle sheath (Cook Medical) was advanced to the ascending aorta, and a 5-F Diagnostic Amplatz Left (AL)-1 catheter (Merit Medical OEM) was then advanced inside of the shuttle sheath to the aortic root.

Using a straight glidewire through the AL-1 catheter, the aortic valve was crossed, and the shuttle sheath was then advanced into the LV (Video 4, Video 5, Video 6). The glidewire was passed through the device mesh in order to center the sheath over the device to facilitate grasping of the device without movement. A rat tooth/alligator grasping forceps (Boston Scientific) within the shuttle sheath was used to grip the Watchman, and the device was retrieved to the descending aorta (Video 7). At this time, another rat tooth/alligator grasping forceps, inserted through the Cook sheath, was used to secure the device at 2 separate points for stability before retrieval into the sheath (Video 8). The Watchman device was then pulled into the sheath and out of the body (Video 9).

An aortogram was performed that did not demonstrate dissection or extravasation (Video 10). Perclose devices (Abbott Cardiovascular) were deployed at the arterial access site. Completion ultrasound was performed on the femoral artery and demonstrated successful hemostasis, good flow, and no significant stenosis. The patient was transferred to the recovery area for observation.

## Outcome and Follow-Up

The patient tolerated the procedure well, and no postoperative complications were noted. Postoperative echocardiogram the following day demonstrated no structural cardiac damage and no pericardial effusion, along with unchanged from prior moderate aortic stenosis and moderate aortic regurgitation.

## Discussion

LAAO devices, including the Watchman device, are elective therapy indicated to reduce the risk of thromboembolism from the LAA in patients with nonvalvular atrial fibrillation in patients who are not candidates for long-term anticoagulation. Based on available reports, LAAO device embolization has been noted to occur in up to 2% of cases, with reported Watchman 2.5 embolization rates of 0.6% and 0.7% in the PROTECT-AF (Watchman Left Atrial Appendage System for Embolic Protection in Patients with Atrial Fibrillation) and PREVAIL (Evaluation of the WATCHMAN Left Atrial Appendage [LAA] Closure Device in Patients With Atrial Fibrillation Versus Long Term Warfarin Therapy) studies, respectively.^2^, ^3^, ^4^ Embolization can lead to serious complications, including obstructive cardiopulmonary shock.^5^ Factors associated with device embolization include undersized occlusion device, occlusion device oversizing, high LAA velocity, low LA pressure or an underfilled LAA at the time of implantation resulting in device undersizing, LAA with cactus shape, and a wide LAA neck.^2^ Both undersizing and oversizing can lead to device embolization via retraction of the anchors due to overcompression, though these occur through different mechanisms. Though data on embolization are limited, a systematic review by Aminian et al^1^ reported that the final anatomical position of LAAO device embolization was most commonly observed in the aorta and LV. In this same systematic review, it was also demonstrated that aortic embolization is most commonly managed with percutaneous intervention, whereas LV embolization is commonly treated with surgical intervention, thus increasing surgical morbidity.^1^ Conventionally, this discrepancy may be explained by concerns that attempts at percutaneous retrieval of LV embolized LAAO devices could result in significant valvular damage due to large-bore sheaths, device instrumentation, and direct trauma from the interaction of the device with the valvular apparatus at the time of extraction.

Currently, few reports of percutaneous intervention exist for Watchman embolization to the LV or LVOT. Here, we describe a novel technique of percutaneous removal using a rat tooth/alligator grasping forceps for extraction from the LVOT after in vitro simulation with an excellent outcome. This particular patient had a history of CABG, making surgical excision significantly more difficult due to the complications associated with redo sternotomy. Rat tooth/alligator grasping forceps have been utilized for Watchman retrieval from the LA, LV cavity, and aorta.^6^, ^7^, ^8^, ^9^ To our knowledge, no cases have been reported employing this tool for LVOT device retrieval. Such cases require multidisciplinary team preplanning in case of complications requiring vascular surgery, transcatheter valve intervention, or emergency cardiac surgery. Furthermore, this case underlines the necessity of routine 6-week follow-up screening TEE, as late embolization is a possibility with significant possible morbidity.^1^

## Conclusions

This novel transaortic percutaneous LAAO device retrieval approach using the rat tooth/alligator grasping forceps provides a safe and effective method to remove LAAO devices from the LVOT without surgical intervention. This approach may be applied with particular utility in patients deemed high surgical risk, including those with prior sternotomy.

## Funding Support and Author Disclosures

Dr D'Avila has received speaker/consulting honoraria from Abbott, Inc, Biosense Webster, Inc, and Biotronik; and has received research support from Medtronic, Inc, Abbott, Inc, Circa Scientific, Inc, and Biosense Webster, Inc. Dr Locke is a consultant for Biosense Webster and Abbott. Dr Laham is a consultant for Medtronic, Edwards Lifesciences, and Abbott. All other authors have reported that they have no relationships relevant to the contents of this paper to disclose.

## Appendix

Video 1 Outside hospital transesophageal echocardiography at time of Watchman placement demonstrating well seated 24 mm Watchman FLX Device within the LA in the left atrial appendage.

Video 2 Bench top demonstration of Watchman being pulled through stenotic aortic valve.

Video 3 Intraoperative transesophageal echocardiography demonstrating Watchman in left ventricular outflow tract with color Doppler.

Video 4 Fluoroscopy demonstrating glidewire being passed through the device mesh in order to center the sheath over the device to facilitate grasping of the device without movement.

Video 5 Intraoperative transesophageal echocardiography demonstrating Watchman with glidewire advanced inside.

Video 6 Fluoroscopy demonstrating the aortic valve being crossed by shuttle sheath using a straight glide wire.

Video 7 Fluoroscopy demonstrating rat tooth/alligator grasping forceps being advanced into the sheath, grasping the Watchman, and extracting it through the aortic valve.

Video 8 Fluoroscopy demonstrating two rat tooth/alligator grasping forceps being used to grasp the Watchman in the descending aorta and pulling it into the Cook Sheath.

Video 9 Intraoperative transesophageal echocardiography demonstrating aortic valve immediately after Watchman removal with color Doppler.

Video 10 Fluoroscopy demonstrating intraoperative aortogram performed that did not demonstrate any complications.
